# Calculation of AeroMACS Spectrum Requirements Based on Traffic Simulator

**DOI:** 10.3390/s21103343

**Published:** 2021-05-11

**Authors:** Hong-Gi Shin, Hyung-Jung Kim, Sang-Wook Lee, Hyun-Goo Yoon, Yong-Hoon Choi

**Affiliations:** 1 NEOWIZ Corp. 14, Daewangpangyo-ro 645beon-gil, Bundang-gu, Seongnam-si 13487, Korea; ghdrl95@neowiz.com; 2 Electronics and Telecommunications Research Institute (ETRI), 218, Gajeong-ro, Yuseong-gu, Daejeon 34129, Korea; acekim@etri.re.kr; 3 RCAST (Research Center for Advanced Science and Technology), The University of Tokyo, Tokyo 153-8904, Japan; sadaota@solab.rcast.u-tokyo.ac.jp; 4 Department of Electronic Engineering, Myongji College, 134, Gajwa-ro, Seodaemun-gu, Seoul 03656, Korea; hgyoon@mjc.ac.kr; 5 School of Robotics, Kwangwoon University, 20, Kwangwoon-ro, Nowon-gu, Seoul 01897, Korea

**Keywords:** AeroMACS, spectrum requirement, airport traffic model, ITU-R M.1768-1, service categories

## Abstract

In this paper, we propose a methodology for calculating the necessary spectrum requirements of aeronautical mobile airport communication system (AeroMACS) to provide various airport communication services. To accurately calculate the spectrum requirement, it is necessary to evaluate the AeroMACS traffic demand of the peak time and statistical data on the packet traffic generated at the airport. Because there is no AeroMACS traffic model and real trace data, we have developed the AeroMACS traffic simulator based on the report of Single European Sky Air Traffic Management Research (SESAR). To calculate the spectrum requirements, the AeroMACS traffic simulator is combined with the methodology of ITU-R M.1768-1. The developed traffic simulator reflects AeroMACS traffic priorities and can generate the required traffic according to its location in the airport. We observed the spectrum requirement by changing the number of sectors and the spectral efficiency. To show the feasibility of our methodology, we applied it to the case of Incheon International Airport in Korea. The simulation results show that the average bandwidth of 0.94 MHz is required in the ground area and 8.59 MHz is required in the entire airport.

## 1. Introduction

Aeronautical mobile airport communication system (AeroMACS) has been proposed to support the increasing need for data communications and information sharing on the airport surface for both fixed and mobile applications [1,2,3,4,5,6,7]. Based on the mature worldwide interoperability for microwave access (WiMAX) standard, AeroMACS operates in the protected and licensed aviation spectrum band from 5091 to 5150 MHz. However, it is necessary to calculate the spectrum requirements considering various operational scenarios, because the required spectrum amount may vary greatly depending on the size of the airport, the location in the airport, and the density of air traffic. AeroMACS enables ground-to-aircraft communications to support not only current applications but also new services that may require more bandwidth.

AeroMACS is a key technology to the aviation industry to improve communications on the airport surface by providing increased transmission of air traffic control (ATC) and airline operations communications (AOC) to relieve traffic, congestions, and delays, and to support the safety and regularity of flight. Since 2007, more than 40 airports worldwide have implemented or are in the process of implementing AeroMACS deployments. In the United States and Europe, AeroMACS services are planned around 2025. For effective operation of the AeroMACS system, which can meet the demand of the latest aviation data traffic, it is necessary to calculate accurate spectrum requirements.

Several recent AeroMACS deployment case studies can be observed. Study [4] is the first attempt to apply AeroMACS to a real airport environment and provides an example of a virtual AeroMACS implementation at Barajas Madrid Airport in Spain and Toulouse Airport in France. Recently, there have been studies on implementing AeroMACS systems and performing performance analysis at Hongqiao [8] and Guilin [9] airports in China. These two studies confirmed that proper base station layout and cell planning are critical to satisfying quality of service (QoS) when AeroMACS systems are deployed at airports. Korowajczuk [10,11] made detailed comments on overall AeroMACS network design issues, and applied this system to Logan Airport in Boston, United States. In this study, data volumes at runway, taxiway, and gate were estimated, and network capacity according to traffic demand was mentioned. Morioka et al. [12,13] deployed an AeroMACS system at Sendai Airport in Japan and showed that the system can effectively meet the communication QoS requirements between the aircraft and the ground. These studies [8,9,10,11,12,13] performed cell planning by estimating the total amount of traffic required for each region in the airport. However, consideration of the priority of the air traffic message was insufficient, and the spectrum requirement was not calculated by a standardized procedure following the recommendation of [14] or [15].

Research on the core technology of the AeroMACS system has also been conducted. Naganawa et al. [16] reported the results of handover tests performed at Sendai Airport, Japan (e.g., latency, round trip time, and received signal strength). A software defined networking (SDN)-based aeronautical communication system architecture has been proposed in work [17]. In this work, performance comparisons have been carried out between the different mobility protocols, LISP-MN and MIPv6/SHIM6, in terms of handover signaling delay for both SDN-based and non-SDN-based architecture. Security-related issues were addressed in studies [18,19,20]. Among the studies on physical layer technology, adaptive modulation-coding (AMC) scheme was covered in [21]. Zolanvari et al. [22] presented a theoretical analysis of the orthogonal frequency-division multiple access (OFDMA) structure of the AeroMACS physical layer. Study [23] proposed the use of a vertical array configuration in base stations to mitigate ground reflection fading for airport surface communications.

Typical spectrum requirements calculation methodologies predict traffic demand based on market research or traffic models and convert it into spectrum requirement. As representative methodologies of spectrum requirement calculation, there is a recommendation ITU-R M.1390 [14] suitable for circuit-switched network and a recommendation ITU-R M.1768-1 [15] suitable for packet-switched network. The former computes the traffic demand with simple Erlang-B formula and the latter computes the traffic demand based on the M/G/1 queuing model, which can reflect statistical characteristics of packet and priorities of service classes. However, both methodologies commonly require traffic data of the wireless system from market research or a traffic model. In the case of international mobile telecommunication (IMT), which has many users all over the world, it is possible to obtain traffic data through market research, while it is difficult to obtain traffic data through market research in some applications, such as AeroMACS, which has relatively fewer users and limited usage. Therefore, we propose a combined methodology for calculating the necessary spectrum requirements of AeroMACS without market research or traffic models.

We have developed an AeroMACS packet traffic simulator based on the research from Single European Sky Air Traffic Management Research (SESAR) and it is combined with the methodology of ITU-R M.1768-1 [15]. To reflect statistical characteristics of packet and priorities of AeroMACS service categories, the input stages of [15] are much changed. We use four traffic categories defined by the International Civil Aviation Organization (ICAO): network management (NET), ATC, AOC, and ground vehicle (VC). The spectrum requirements are observed by varying the size of the airports classified by the number of aircraft processed in one hour, the operational domains (i.e., ramp, ground, and tower), and spectral efficiency which is related to service coverage called cell or sector. In general, spectral efficiency is inversely proportional to the size of sector. Therefore, the spectrum requirements should be calculated by applying appropriate spectral efficiency values to the operational domains. It is found that the highest spectrum is required in ground and ramp area where a relatively large amount of data traffic occurs, and a relatively low spectrum is required in tower area.

The organization of this paper is as follows. Section 2 describes the characteristics of the AeroMACS system and the data traffic model of the airport. Section 3 describes how to calculate spectrum requirements. Section 4 describes the results of calculating spectrum requirements for various offered traffic load, areas within the airport, and spectral efficiencies using the method described in Section 3. The traffic generator based on the SESAR studies [24,25] and the implementation details of the spectrum calculator based on the methodology of Rec. ITU-R M.1768-1 [15] are also mentioned. Finally, Section 5 concludes this paper.

## 2. AeroMACS Data Traffic Model

AeroMACS uses wireless broadband technology that supports the increasing need for data communications and information sharing on the airport surface for fixed and mobile applications now and into the future. The mobile nodes are aircraft and ground vehicles. The base station shall be installed in a suitable location to serve the tower area that controls the runway, the ground area that controls the taxiway and the parking area, and the ramp area that controls the gates. The overall network structure of AeroMACS system is described in detail in studies European Organization or Civil Aviation Equipment (EUROCAE) [2] and WiMAX Forum [4].

The AeroMACS data link shall be able to support airport communication services while the subscriber speed is lower or equal to 50 knots (92.6 km/h). AeroMACS is used from the time when the aircraft enters the airport, and the speed is reduced to 50 knots or less. When an aircraft arrives at the airport, it uses a variety of airport communication services according to a set of procedures until it takes off. The communication services provided in each area are categorized as NET, ATC, AOC, and VC, and have detailed services for each service category as shown in Table 1. Each of the detailed services has different QoS requirements. Table 1 summarizes all air communication services described in [4] from aircraft landing to ground stay and takeoff. The vertical direction in Table 1 is arranged according to flow of time, and the horizontal direction is arranged according to the priority of the service. See pages 61 to 65 of Reference [4] for the abbreviation and meaning of the message.

As shown in Table 1, the most services are provided in the ramp area at the departure phase. A detailed description of each service can be found in EUROCAE document [4]. SESAR [24] performed similar classification and has an additional service category, which is VC. A noticeable difference between studies [4,24] is the phase and operational domain where detailed service is performed. In study [24], the electronics flight folder update (EFFU) and the flight operational quality assurance (FOQA) that cause the largest amount of traffic are classified as being performed at the arrival phase. The EFFU service is performed in ground area, and the FOQA service is performed in the ramp area of the arrival scenario, which accounts for approximately 87% of the total traffic. In this paper, experiments were performed according to the classification of SESAR [24].

The traffic pattern and demand for each AeroMACS service scenario were studied in SESAR [24,25,26]. Traffic for each service was generated by simulations in consideration of the area within the airport, the occurrence frequency, the residence time of each area of the aircraft, the number of aircraft arrivals and departures per hour, and the average number of aircraft within an airport. A total of 32 service scenarios were considered. Each scenario was based on the average number of aircraft handled per hour at the airport, the spatial classification (i.e., tower, ground, or ramp), and the temporal classification (i.e., ARRIVAL or DEPARTURE). ARRIVAL is a scenario where the arrival rate of an aircraft is twice the departure rate, and DEPARTURE is the opposite scenario. The service scenarios and offered traffic loads used in this paper are described in Section 4.

Gheorghisor et al. [27,28] calculated the offered traffic load through Monte Carlo simulations for various scenarios in which there were 200 aircraft, 40 ground vehicles, and 40 sensors. The derived offered load was used as input for the newly developed AeroMACS model based on the Riverbed Modeler Tool, and the spectrum planning of the Dallas–Fort Worth airport was performed through simulation.

## 3. Proposed Methodology

**Figure 1:**
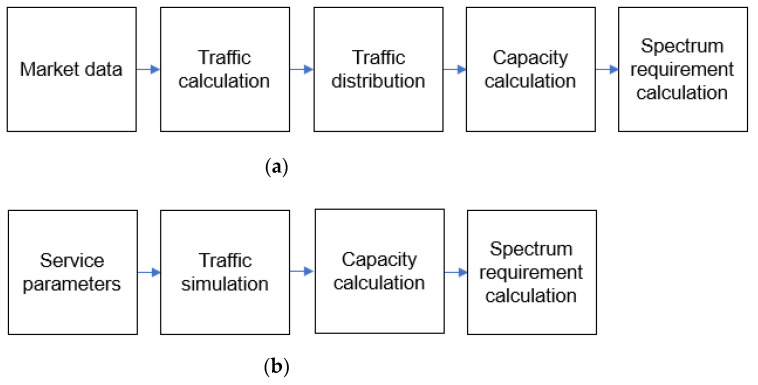
General flow and proposed flow of spectrum requirement calculation for AeroMACS; (**a**) General flow of spectrum requirement calculation; (**b**) Proposed flow of spectrum requirement calculation.

Generally, the technical process of estimating spectrum requirements for mobile communications is based on four essential issues: definition of services, market expectations, technical and operational framework, and spectrum calculation algorithm. The conventional methodology in ITU-R M.1768-1 [15] consists of several steps starting with market data analysis. It uses the parameters obtained from market data analysis in the Poisson traffic model. Then, it distributes traffic to several radio environments to determine required system capacity to carry the offered traffic. Capacity calculation algorithms are given separately for circuit-switched and packet-switched service categories. Finally, it calculates the spectrum requirements and applies necessary adjustments to consider practical network deployments. The overall flow is shown in Figure 1a.

There is much market data analysis available for mobile communications such as LTE, whereas it is difficult to obtain data traffic model or real traffic trace for AeroMACS. Therefore, we propose a combined methodology which substitutes traffic calculation and distribution blocks in Figure 1a into the traffic simulation block shown in Figure 1b to estimate the required spectrum of a wireless service without market analysis. The traffic simulation block derives offered traffic load and packet generation probabilities for each service at a given airport. Then, its output is used as the input to the capacity calculation block as shown in Figure 1b.

### 3.1. Traffic Simulation

**Figure 2:**
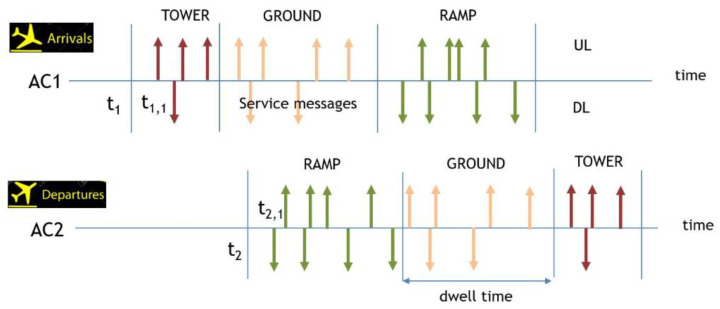
Possible message trigger events in an airport.

AeroMACS services begin as the aircraft enters the airport, and the service follows predetermined procedures based on the aircraft’s location as shown in Figure 2. The arriving aircraft AC1 moves in the order of tower, ground, ramp, while departing aircraft AC2 moves in the order of ramp, ground, tower. The *k*th message generation time of AC1 is denoted by $t_{1,k}$. The subscript *k* indicates one of the AeroMACS messages given in Table 1. Up arrow indicates uplink (UL) message and down arrow indicates downlink (DL) message. As it is generally accepted that the message generation time *t* is a random process determined by the operational domain and flight phase, we develop a stochastic data traffic simulator based on the parameters reported in [4,24,25,26]. The following assumptions are used in this paper.

- The number of aircraft in the airport should be a Poisson distribution.
- Each arrival and departure phase length (in seconds) should be a uniform distribution ±10% of the mean value.
- Arrival and departure rates are an exponential distribution to model the aircraft arrival and departure processes.
- The message generation time, $t_{n,k}$, denoted by the *k*th message generation time of the *n*th aircraft should be a uniform distribution.

**Figure 3:**
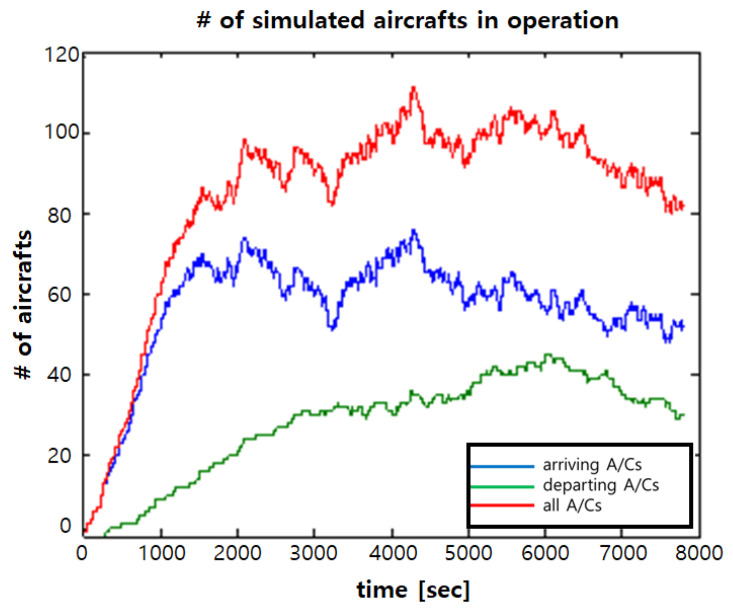
Number of aircraft over time (average number of aircraft = 100, y=0.2x).

**Figure 4:**
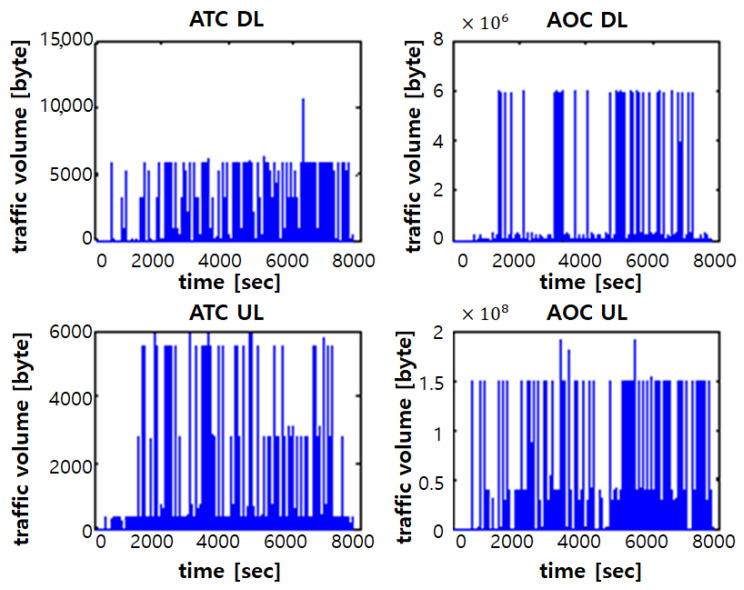
Traffic volume samples of service messages at the ramp area.

The flow of the traffic simulation is as follows. First, a number of ACs with a given average value is generated as a Poisson random process according to the given scenario and phase. Next, AeroMACS service start time of each aircraft and the dwell time for each area are generated as a uniform random process in time. Then, message generation time in each airport area is determined with a uniform distribution random process. The used settings and parameter values for generating each service message in the traffic simulation are given in Figure A1 of [25]. Finally, this procedure is applied to all ACs. Figure 3 shows the simulated number of ACs over time when the average AC number in processing is 100 and the relationship of the arrival rate of arriving aircraft (say *x*) and the arrival rate of departing aircraft (say *y*) is y=0.2x, which results in 66.55 arriving ACs and 33.45 departing ACs. The simulated traffic load of ATC and AOC generated at the ramp area from all ACs are shown in Figure 4.

### 3.2. Calculation of Required System Capacity

After traffic simulation described in the previous section, the system capacity to satisfy all four service categories (i.e., NET, ATC, AOC, and VC) with different packet length, priority, and average delay should be calculated. Then, the capacity is converted to the spectrum requirement using the spectral efficiency suited to the radio environment. Because there is insufficient data traffic model available for the AeroMACS system and real trace data is not disclosed, we assumed that the arrival times of packets are independent of each other, have exponential distribution, and that the length of packet has arbitrary distribution. In addition, it was assumed that the packet processing is performed on a first come first serve (FCFS) basis within a single queue and is served with non-preemptive priority, and the system capacity is measured using the M/G/1, respectively. To calculate the system capacity using the M/G/1 model, the following input parameters must be determined:

- $T_{n}\left(bit/s/cell\right)$: the offered load for service category (SC) *n*.
- $s_{n}\left(bit/packet\right)$ and $s_{n}^{\left(2\right)}$: The mean and the second moment of the IP packet size distribution of each SC.
- The required mean delay $D_{n}$ of each SC.
- The priority ranking of all service categories *n* with n=1, 2,…, N. It is assumed that the service category n=1 has the highest priority.

The resulting IP packet arrival rate per cell $\lambda_{n}\left(packets/s/cell\right)$ of service category *n* is obtained by dividing the offered traffic by the mean packet size:

(1) $$\lambda_{n}=\frac{T_{n}}{s_{n}}.$$

The aggregated arrival rate over all SCs is denoted by:

(2) $$\lambda =\overset{N}{\underset{n=1}{\sum }}\lambda_{n}.\;$$

The system capacity $C_{n}$ that is needed to obtain the mean delay required by SC *n* can be calculated in the following procedure. The priority level requiring the highest capacity denotes the total required system capacity, since for the case in which the QoS requirements of the most demanding service category are fulfilled, the requirements of the other service categories are over-fulfilled. Therefore, the overall required system capacity is given by:

(3) $$C=\max\left(C_{1},\;C_{2},\;\ldots ,C_{n}\right).$$

The average delay $D_{n}$ of the IP packets of SC *n* is the sum of the average waiting time and the average service time. When the queue is FCFS and the server uses a non-preemptive priority service, $D_{n}$ can be calculated with the Cobham formula [29]:

(4) $$D_{n}\left(C_{n}\right)=\left[\overset{N}{\underset{i=1}{\sum }}\lambda_{i}s_{i}^{\left(2\right)}/\left(2\left(C_{n}-\overset{n}{\underset{i=1}{\sum }}\lambda_{i}s_{i}\right)\left(C_{n}-\overset{n-1}{\underset{i=1}{\sum }}\lambda_{i}s_{i}\right)\right)\right]+\frac{s_{n}}{C_{n}}.$$

The system capacity $C_{n}$ satisfying $C_{n}=D_{n}\left(C_{n}\right)$ can be obtained from Equation (3) and is a solution of the cubic equation:

(5) $$a_{n}x^{3}+b_{n}x^{2}+c_{n}x+d_{n}=0.$$

The coefficients $a_{n}$, $b_{n}$, $c_{n}$, and $d_{n}$ are expressed by:

(6) $$a_{n}=2D_{n},$$

(7) $$b_{n}=-2\left(D_{n}\left(\overset{n}{\underset{i=1}{\sum }}\lambda_{i}s_{i}+\overset{n-1}{\underset{i=1}{\sum }}\lambda_{i}s_{i}\right)+s_{n}\right),\;$$

(8) $$c_{n}=2\left(D_{n}\left(\overset{n}{\underset{i=1}{\sum }}\lambda_{i}s_{i}\right)\left(\overset{n-1}{\underset{i=1}{\sum }}\lambda_{i}s_{i}\right)+s_{n}\left(\overset{n}{\underset{i=1}{\sum }}\lambda_{i}s_{i}+\overset{n-1}{\underset{i=1}{\sum }}\lambda_{i}s_{i}\right)\right)-\overset{N}{\underset{i=1}{\sum }}\lambda_{i}s_{i}^{\left(2\right)},$$

(9) $$d_{n}=-2s_{n}\left(\overset{n}{\underset{i=1}{\sum }}\lambda_{i}s_{i}\right)\left(\overset{n-1}{\underset{i=1}{\sum }}\lambda_{i}s_{i}\right).\;$$

For the solution of cubic equations, a good symbolic solution is available by using Cardano’s formula. Mathematically, Equation (5) has three solutions. To determine the correct solution among these three solutions, the stability border of the queuing system should be considered, i.e.:

(10) $$\overset{n}{\underset{i=1}{\sum }}\lambda_{i}s_{i}<C_{n}.$$

The spectrum requirement *F* can be derived based on the system capacity obtained from Equation (3) and the spectrum efficiency η (bit/s/Hz) by:

(11) $$F=\frac{C}{\eta }$$

where *C* is the sum of the uplink and downlink capacity. In this paper, we have tested only two service categories: ATC and AOC, since the NET and VC traffic is relatively small and has little effect on the spectrum requirements.

## 4. Simulation Study

### 4.1. Simulation Scenario

In this paper, we selected the scenario most similar to Incheon International Airport (ICN) among the scenarios in [24] in order to estimate the required spectrum of the AeroMACS system at ICN airport in Korea. The average number of flights on the ICN during peak hour is 58.77, which is similar to the scenario of operating an average of 50 aircraft in SESAR’s traffic simulation.

As shown in Table 2, the offered load is different according to operational domain, flight phase of an aircraft, service category, and direction of data transmission (UL/DL). According to [24], traffic is stochastically generated based on the rate of aircraft arrival and departure. There is no restriction on the packet length, and therefore, the fluctuation of the traffic load over time is very large. The arrival scenario assuming an average of 50 aircraft is the result of calculating the offered load assuming that the average number of arriving aircraft is 33.27/h and the average number of departing aircraft is 16.73/h. In the departure scenario, we assumed that the average number of arriving aircraft is 14.23/h and the average number of departing aircraft is 35.77/h. In this paper, system capacity is estimated based on the average traffic load during the peak hour.

In addition to the offered load shown in Table 2, a first and second moment of packet length are required to calculate the system capacity using the M/G/1 queuing model. Table 3 shows the average packet length and Table 4 shows the second moment of packet length used in our experiments. They are calculated for each operational domain, flight phase of an aircraft, service category, and transmission direction. In [24,25], simulation was performed using the same packet length as the message length., In the case of a service with a very large size of a message such as EFFU or FOQA, segmentation into appropriate packets should occur. However, for comparison with SESAR results, the same average packet lengths to [24,25] are used.

References [4,24] show the required latency for each type of service. In this paper, we used the smallest value for each service, 1.2 s for ATC and 3.0 s for AOC. Reference [2] suggests spectral efficiency depending on cell radius and airport size. In this paper, as shown in [2], the maximum radius of cells in the ramp area is set to 0.95 km, and the maximum radius of cells in the tower and ground areas are set to 2.5 km. In calculating the maximum spectral efficiencies, 64QAM for downlink and 16QAM for uplink are used, and the spectral efficiency according to cell coverage are set as shown in Table 5 and Table 6. This is based on airports operating more than 50 aircraft per hour.

Packets are assumed to be evenly distributed in each specific operational domain. Since the detailed layout in the airport is not considered, the directions of the runways, the number of gates, terminal shape and arrangement are not taken into account.

**Figure 5:**
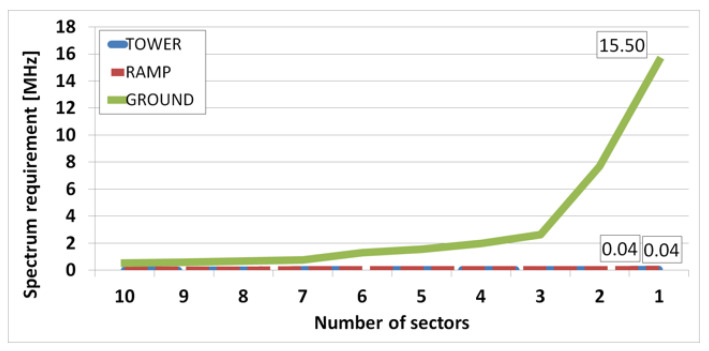
Spectrum requirement according to the number of sectors in arrival scenario.

**Figure 6:**
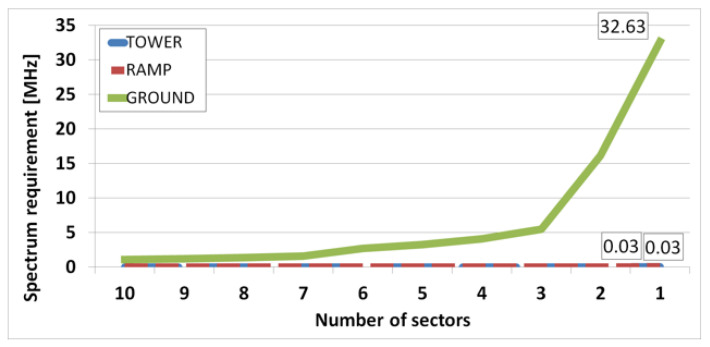
Spectrum requirement according to the number of sectors in departure scenario.

We performed two types of experiments. First, the spectrum requirements of ATC and AOC service were calculated according to the number of sectors, which are shown in Figure 5 and Figure 6. Figure 5 shows the experimental results for the arrival scenario, and Figure 6 shows the experimental results for the departure scenario. The spectrum requirement is the sum of the spectrum requirements for downlink and uplink.

Secondly, we calculated the total spectrum requirement according to offered traffic load at ICN airport. In this experiment, the total number of sectors and the cell radius of ICN airport are assumed as shown in Table 7. This is roughly derived from the size of ICN airport, runway length, and gate configuration. The ground area deploys several cells because it needs to handle large area traffic. Table 7 summarizes the experiment environment of ICN airport by applying the spectral efficiency according to Table 5 and Table 6. Table 8 shows the results of the spectrum requirements for the arrival scenario and departure scenarios.

### 4.2. Results and Discussion

Figure 5 is a graph of the spectrum requirements according to the number of sectors in the arrival scenario. When all the traffic from the entire airport is served by one base station sector, spectrum requirements of 0.04 MHz for both tower and ramp areas and 15.5 MHz for ground area are obtained. It is noted that using one sector requires four 5 MHz channels. However, because of the limited size of the sector, it is necessary to use multiple sectors to cover the entire airport.

Figure 6 is a graph of the spectrum requirements according to the number of sectors in the departure scenario. When all the traffic from the entire airport is served by one base station sector, the spectrum requirements of 0.03 MHz for both tower and ramp areas and 32.63 MHz for ground area are calculated. The reason why the ground area has a quite large spectrum requirement is that it includes EFFU services that make up more than 90% of the total traffic. Therefore, to save spectral resources, it is necessary to regularly perform EFFU services in parking areas, not on taxiways. We have clearly seen that ATC applications at the ramp area are not data intensive, regardless of whether an aircraft is arriving or departing. The average offered load is roughly a few kbps. The same happens with AOC applications if we do not consider the three large AOC services: EFF, UPLIB, and E-CHARTS.

The second column of Table 8 shows the spectrum requirements when the AeroMACS cells are deployed at ICN airport in the arrival scenario. Based on the average traffic, it was observed that a spectrum band of up to 0.941 MHz per sector is required in the ground area. The spectrum band of 8.585 MHz (ramp: 0.017 MHz/sector × 6, tower: 0.005 MHz/sector × 1, ground: 0.942 MHz/sector × 9) is required for the entire airport if frequency reuse is not considered. The experiment was carried out assuming that EFFU was performed in the ground area upon arrival. If the EFFU service is performed in a separate parking area, it is expected that spectrum resources will be used more efficiently.

The third column of Table 8 shows the spectrum requirements when cells are deployed at ICN airport in the departure scenario. The highest spectrum requirement per sector during average traffic was calculated to be 1.89 MHz in the ground area. Compared with the second column of Table 8, the arrival aircraft has higher traffic load in the ground area than the departing aircraft, and the traffic load in the ramp area is lower.

In this experiment, only two service categories were considered: ATC and AOC, but NET and VC still exist in the airport communication service category. NET is the highest priority service category, but the traffic load is very low, VC is the lowest priority category, and traffic load is also very low compared to AOC. Therefore, these two service categories were not considered in calculating the spectrum requirements. Radio parameters are the most important factor in calculating the spectrum requirements, but they are affected by the location of the base stations and the direction of the antenna. For example, the operator can increase the BS antenna down tilt to concentrate power near the gate in the ramp area. On the other hand, to achieve maximum coverage, the BS antenna slope can be set to an appropriate level. Thus, a trade-off will be necessary for each airport.

## 5. Conclusions

In this paper, we propose a combined methodology for simulating an AeroMACS traffic pattern and estimating the necessary spectrum requirements of AeroMACS without market research or traffic model. To accurately calculate the spectrum requirement, it is necessary to evaluate the AeroMACS traffic demand of the peak time and statistical data on the packet traffic generated at an airport. Because there is no AeroMACS traffic model and real trace data, we have developed the AeroMACS traffic simulator based on the reports of SESAR and EUROCAE. The spectrum requirement of AeroMACS was then derived from the standard methodology described in ITU-R M.1768-1 using M/G/1 queuing model. The developed traffic simulator reflects AeroMACS traffic priorities and can generate the required traffic according to its location in the airport. We observed the spectrum requirement by changing the operational domain, the size and the number of sectors, and the spectral efficiency. Experiments conducted at ICN airport required 0.94 MHz per sector in the ground area based on the average traffic load in arrival scenario and 1.89 MHz per sector based on the average load in departure scenario. Given the actual traffic data of AreoMACS, we expect that the statistical parameters used in the traffic simulator can be modified to fit the actual traffic data, so that the accuracy of the spectrum requirement prediction can be improved. Therefore, it can be concluded that the method proposed in this paper can be widely applied to predict the service data traffic pattern of a new radio link and more accurately estimate spectrum requirements on condition that actual traffic data are given.

## Figures and Tables

**Table 1:** AeroMACS services executed during arrival and departure phase.

Phase	Area	Services		
		NET	ATC	AOC
Arrival	Tower	NETKEEP	ACM	OOOI, AUTOLAND-REG
	Ground		SURV, ACL, D-SIG, D-TAXI	EFFU, FLT-JOURNAL, TECHLOG, CREW-TIME
	Ramp		ACM	OOOI, FOQA, FLTLOG, CABINLOG, ETS-REPORT, REFUEL
Departure	Ramp	NETCONN, NETKEEP	DLL, COTRAC, DOTIS, D-SIGMET, DCL, FLIPCY, FLIPINT, D-RVR, D-SIG, D-FLUP, PPD, D-TAXI	AOCDLL, LOADSHT, E-CHARTS, UPLIB, SWCONF, SWLOAD25, SWLOAD, BRFCD, ACLOG, TECHLOG, AIRWORTH, WXTEXT, PASSENGER, CREW-RPS, CREW-BUL, CREW-REG, FLTPLAN, NOTAM, EFF, WXGRAPH, CREW-L, HANDLING, CATERING, BAGGAGE, NOTOC, LOADDOC, PREFLT-INS, DOOR, FLOWCON, EFFU, TAKEOFF-CALC, OOOI
	Ground		SURV, ACL, ACM	
	Tower		ACM	WXRT, OOOI

**Table 2:** Traffic when an average of 50 aircraft are in operation.

Area	SC	DL Traffic Volume [Kbps]		UL Traffic Volume [Kbps]	
		Arrival	Departure	Arrival	Departure
Tower	ATC	0	0	0.13	0.09
	AOC	2.69	2.01	0.06	0.12
Ramp	ATC	1.68	1.18	0.92	0.64
	AOC	1.10	0.67	0.14	0.15
Ground	ATC	0.41	0.86	0.35	0.58
	AOC	163.94	336.00	7691.73	16,280.31

**Table 3:** Average packet length according to areas, flight phase of an aircraft, service categories, and transmission directions.

Area		Tower		Ramp		Ground	
SC		ATC	AOC	ATC	AOC	ATC	AOC
Arrival	DL [Kbps]	0.034	0	0.282	0.522	0.034	0.086
	UL [Kbps]	0.123	0.522	0.241	0.299	0.149	0.523
Departure	DL [Kbps]	0.034	0	0.307	0.523	0.035	0.086
	UL [Kbps]	0.123	0.523	0.234	0.501	0.149	0.523

**Table 4:** The second moment of packet length according to areas, flight phase of an aircraft, service categories, and transmission directions.

Area		Tower		Ramp		Ground	
SC		ATC	AOC	ATC	AOC	ATC	AOC
Arrival	DL [Kbps]	1.175	0	120.67	279.68	1.20	7.56
	UL [Kbps]	15.504	279.74	91.60	104.96	23.43	280.56
Departure	DL [Kbps]	1.175	0	135.80	280.50	1.39	7.56
	UL [Kbps]	15.504	280.29	89.52	262.91	23.42	280.56

**Table 5:** Spectral efficiency according to ramp area coverage.

	Maximum Coverage [%]	Spectral Efficiency [bps/Hz]
UL	≤25	3
	≤37.5	2
	≤50	1.5
	≤70	1
	≤100	0.5
DL	≤22.5	4.5
	≤37.5	4
	≤45	3
	≤67.5	2
	≤90	1.5
	≤100	1

**Table 6:** Spectral efficiency according to tower and ground area coverage.

	Maximum Coverage [%]	Spectral Efficiency [bps/Hz]
UL	≤15	3
	≤27.5	2
	≤40	1.5
	≤62.5	1
	≤100	0.5
DL	≤7.5	4.5
	≤10	4
	≤17.5	3
	≤32.5	2
	≤47.5	1.5
	≤77.5	1

**Table 7:** Sector planning and experimental environment at ICN airport.

Area	Ramp	Tower	Ground
Number of sectors	3	1	3
Number of sectors per site	2	1	3
Total number of sectors	6	1	9
Cell radius [km]	0.7	2.5	1.44
Maximum coverage area [%]	73.7	100	57.7
DL Spectral efficiency [bps/Hz]	0.5	0.5	1.0
UL Spectral efficiency [bps/Hz]	1.5	0.5	1.0

**Table 8:** Simulation results for ICN airport in arrival and departure scenarios.

Area	Spectrum Requirement [MHz]	
	Arrival	Departure
Tower	0.00512	0.00685
Ramp	0.01694	0.01153
Ground	0.94152	1.89747

## Data Availability

The data presented in this study are available on request from the authors.
